# Pathology in General Practice

**Published:** 1907-03-09

**Authors:** 


### PATHOLOGY IN GENERAL PRACTICE.

### "The Demonstration of Tubercle Bacilli."

Of all the ordinary clinical bacteriological tests perhaps none is of greater importance to the general practitioner than the demonstration of the tubercle bacillus. Fortunately the technique is so simple and easy of application that it lies well within the powers of the most ordinary individual safely to accomplish it. Little is required in the way of apparatus, and there is therefore no very definite reason why every practitioner should not have some small room in his house fitted up with some of the commoner stains and appliances for this, and some of the other tests, urinary or bacteriological. Of course, a microscope is an essential, and this preferably should have a mechanical stage. It must also be fitted with an Abbe condenser, an iris diaphragm, three objectives, a $\frac{2}{3}$ in., a $\frac{1}{6}$ in., and a $\frac{1}{12}$ in. oil immersion, and should have a low and a high eye-piece. In addition to this, slides, cover-glasses, a platinum needle, a spirit lamp, a tap of running water with a sink (if possible), and the different stains will be required. The whole outfit, apart from the microscope, will cost very little (one or two pounds), and it may conveniently be placed along with and kept in the same room as the reagents required for testing the urine. Special little glass-stoppered drop bottles should be used for keeping the different stains in, and the latter can always be bought ready-made up from any of the dealers, thus doing away with the difficulty of having weights-and-measure glasses. Most stains also, it must be remembered, require filtering before use, and a few little glass funnels with some filter paper should always be kept at hand.

Having then fitted up such a small laboratory (the requisites for which having been obtained from Baird and Tatlock, Hatton Garden, or any other maker), one may then test the different secretions of the body for the tubercle bacillus. This organism, with leprosy, and some others are called acid fast. They are very difficult to stain with any of the simple watery solutions of the anilin dyes, but when acted on by a powerful stain containing a mordant, especially if this be prolonged or if its action be aided by heat, then they stain easily, and, once having done so, resist very strongly decolorising agents such as the mineral acids. Any combination of gentian violet or fuchsin with anilin oil or carbolic acid may be used, but the procedure which goes by the name of Ziehl-Nielsen's method is the one most usually employed. For this we require carbol-fuchsin, which may be made up as follows:—

| | |
|---|---|
| Basic fuchsin ... ... ... ... | 1 part |
| Absolute alcohol ... ... ... ... | 10 parts |
| Solution of carbolic acid (1-20) ... | 100 parts |

or

| | |
|---|---|
| Sat. alcoholic of fuchsin ... ... ... | 10 parts |
| 5 per cent. sol. of carbolic acid ... ... | 90 parts |

then some mineral acid, 25 per cent. nitric, sulphuric, or hydrochloric, and a counterstain, preferably a blue one, saturated aqueous solution of methylene-blue.

Some prefer to combine the acid and counterstain together, and Fraenkel's modification may be adopted, it having this advantage that only two bottles are required:—

| | |
|---|---|
| Distilled water ... ... ... ... | 50 parts |
| Absolute alcohol ... ... ... ... | 30 parts |
| Nitric acid ... ... ... ... ... | 20 parts |
| Methylene-blue in crystals to saturation. | |

If the sputum of the case is to be examined, one should make the individual, after cleansing out his mouth with some antiseptic to get rid of the common mouth bacilli, spit into a clean glass. With the platinum needle one may then take up a piece of the thickest part and spread it out on a clean glass slide or cover slip, being careful to sterilise the needle after use. After this, one fixes the film by heating over the spirit lamp, taking care not to scorch it, and then pours on the carbol fuchsin. This is to be heated now in turn till bubbles of steam appear (two to three minutes required), and then washed in water. Next apply the 25 per cent. sulphuric acid; and, alternately washing and re-applying the acid, go on until the film has only a very pale pink tint. When this appears wash well; then apply the counterstain (methylene-blue for half a minute), dry, and, if only required for diagnosis, examine without mounting under the oil immersion lens. Tubercle, leprosy, or other acid fast bacilli will be bright red; other bacilli, cocci, pus cells, and the general stroma will be blue, the contrast of the two colours showing up very distinctly. If the specimen is a specially good one and worth keeping, all that is required is to wash the cedar wood oil off with xylol, and then to mount in Canada balsam. If Fraenkel's modification is used the procedure is the same up to the end of the fuchsin staining; then the blue acid stain is applied and kept on until all the red colour disappears and the film becomes blue. Urine and other discharges may be treated in the same way. It is better when dealing with urine to centrifuge first, so as to get a good deposit; then some of this is smeared on a slide, fixed and stained exactly as sputum. It is perhaps a little more difficult to detect the bacilli here, as their numbers may be small; but a careful search should reveal them, generally in little clumps, amongst the pus cells. Pleural fluid, ascitic fluid, and cerebro-spinal fluid may also be examined. Even in cases which prove later at autopsy to be tubercular, bacilli are exceedingly difficult to find, so their absence does not contraindicate tubercle. The treatment of sections varies in several important details from that of the ordinary films we have just been describing. We will therefore refer to them later when we have discussed section-cutting and mounting.

